# On powers that are sums of consecutive like powers

**DOI:** 10.1007/s40993-016-0068-0

**Published:** 2017-02-14

**Authors:** Vandita Patel, Samir Siksek

**Affiliations:** Mathematics Institute, University of Warwick, Coventry, CV4 7AL UK

**Keywords:** Exponential equation, Bernoulli polynomial, Newton polygon, Primary 11D61, Secondary 11B68

## Abstract

Let $k \ge 2$ be even, and let *r* be a non-zero integer. We show that for almost all $d \ge 2$ (in the sense of natural density), the equation

$$x^k+(x+r)^k+\cdots +(x+(d-1)r)^k=y^n,\quad x,~y,~n \in {\mathbb Z}, \; n \ge 2,$$

has no solutions.

## Background

The problem of cubes that are sums of consecutive cubes goes back to Euler ([10] art. 249) who noted the remarkable relation $3^3+4^3+5^3=6^3$. Similar problems were considered by several mathematicians during the nineteenth and early twentieth century as surveyed in Dickson’s *History of the Theory of Numbers* ([7] p. 582–588). These questions are still of interest today. For example, both Cassels [5] and Uchiyama [17] determined the squares that can be written as sums of three consecutive cubes. Stroeker [16] determined all squares that are expressible as the sum of $2 \le d \le 50$ consecutive cubes, using a method based on linear forms in elliptic logarithms. More recently, Bennett, Patel and Siksek [2] determined all perfect powers that are expressible as sums of $2 \le d \le 50$ consecutive cubes, using linear forms in logarithms, sieving and Frey curves. There has been some interest in powers that are sums of *k*-th powers for other exponents *k*. For example, the solutions to the equation

$$\begin{aligned} x^k+(x+1)^k+(x+2)^k=y^n, \quad x,~y,~n \in {\mathbb Z}, \; n \ge 2, \end{aligned}$$

have been determined by Zhongfeng Zhang [18] for $k=2$, 3, 4 and by Bennett, Patel and Siksek [1] for $k=5$, 6, and similar problems are considered by Soydan [15].

In view of the above, it is natural to consider the equation

1 $$\begin{aligned} x^k+(x+1)^k+\cdots +(x+d-1)^k=y^n,\quad x,~y,~n \in {\mathbb Z}, \; n \ge 2 \end{aligned}$$

with *k*, $d \ge 2$. This was studied by Zhang and Bai [19] for $k=2$. They show that if *q* is a prime $\equiv \pm 5 \pmod {12}$ and ${{\mathrm{\upsilon }}}_q(d)=1$ then Eq. (1) has no solutions for $k=2$; here ${{\mathrm{\upsilon }}}_q(d)$ denotes the *q*-adic valuation of *d*. It follows from a standard result in analytic number theory (as we shall see later) that the set of *d* for which there is a solution with $k=2$ has natural density 0. We prove the following generalization to all even exponents *k*.

### Theorem 1

Let $k\ge 2$ be even and let *r* be a non-zero integer. Write $\mathcal {A}_{k,r}$ for the set of integers $d \ge 2$ such that the equation

2 $$\begin{aligned} x^k+(x+r)^k+\cdots +(x+(d-1)r)^k=y^n,\quad x,~y,~n \in {\mathbb Z}, \; n \ge 2, \end{aligned}$$

has a solution (*x*, *y*, *n*). Then $\mathcal {A}_{k,r}$ has natural density 0; by this we mean

$$\begin{aligned} \lim _{X \rightarrow \infty } \frac{ \# \{d \in \mathcal {A}_{k,r} \; :\; d \le X\}}{X} =0. \end{aligned}$$

If *k* is odd, then $\mathcal {A}_{k,r}$ contains all of the odd *d*: we can take $(x,y,n)=(r(1-d)/2,0,n)$. Thus the conclusion of the theorem does not hold for odd *k*.

## Some properties of Bernoulli numbers and polynomials

In this section we summarise some classical properties of Bernoulli numbers and polynomials. These are found in many references, including [8]. The Bernoulli numbers $B_k$ are defined via the expansion

$$\begin{aligned} \frac{x}{e^x-1}=\sum _{k=0}^\infty B_k \frac{x^k}{k!} \, . \end{aligned}$$

The first few Bernoulli numbers are

$$\begin{aligned} B_0=1, \quad B_1=-1/2, \quad B_2=1/6, \quad B_3=0, \quad B_4=-1/30, \quad B_5=0, \quad B_6=1/42. \end{aligned}$$

It is easy to show that $B_{2k+1}=0$ for all $k \ge 1$. The $B_k$ are rational numbers, and the von Staudt–Clausen theorem asserts that for $k \ge 2$ even

$$\begin{aligned} B_k+\sum _{(p-1) \mid k} \frac{1}{p} \; \in \; {\mathbb Z}\end{aligned}$$

where the sum ranges over primes *p* such that $(p-1) \mid k$.

The *k*-th Bernoulli polynomial can be defined by

3 $$\begin{aligned} B_k(x)=\sum _{m=0}^k \left( {\begin{array}{c}k\\ m\end{array}}\right) B_m x^{k-m}. \end{aligned}$$

Thus it is a monic polynomial with rational coefficients, and all primes appearing in the denominators are bounded by $k+1$. It satisfies the symmetry

4 $$\begin{aligned} B_k(1-x)=(-1)^k B_k(x), \end{aligned}$$

the identity

5 $$\begin{aligned} B_k(x+1)-B_k(x)=k x^{k-1}, \end{aligned}$$

and the recurrence

6 $$\begin{aligned} B_k^\prime (x)=k B_{k-1}(x). \end{aligned}$$

Whilst all the above results have been known since at least the nineteenth century, we also make use of the following far more recent and difficult theorem due to Brillhart [3] and Dilcher [9].

### Theorem 2

(Brillhart and Dilcher) The Bernoulli polynomials are squarefree.

### Relation to sums of consecutive like powers

#### Lemma 2.1

Let *r* be a non-zero integer and *k*, $d \ge 1$. Then

$$\begin{aligned} x^k+(x+r)^k+\cdots +(x+r(d-1))^k= \frac{r^k}{k+1} \left( B_{k+1}\left( \frac{x}{r}+d\right) -B_{k+1}\left( \frac{x}{r}\right) \right) . \end{aligned}$$

This formula can be found in ([8] Section 24.4), but is easily deduced from the identity (5).

#### Lemma 2.2

Let $q\ge k+3$ be a prime. Let *a*, *r*, *d* be integers with $d \ge 2$, and $r \ne 0$. Suppose $q \mid d$ and $q \not \mid r$. Then

$$\begin{aligned} a^k+(a+r)^k+\cdots +(a+r(d-1))^k \equiv r^k\cdot d \cdot B_k(a/r) \pmod {q^2}. \end{aligned}$$

#### Proof

By Taylor’s Theorem

$$\begin{aligned} B_{k+1}(x+d)=B_{k+1}(x)+d \cdot B_{k+1}^\prime (x) + \frac{d^2}{2} B_{k+1}^{(2)}(x)+\cdots +\frac{d^{k+2}}{(k+2)!} \cdot B_{k+1}^{(k+2)}(x). \end{aligned}$$

It follows from the assumption $q \ge k+3$ that the coefficients of $B_{k+1}(x)$ are *q*-adic integers. Thus the coefficients of the polynomials $B_{k+1}^{(i)}(x)/{i!}$ are also *q*-adic integers. As $q \mid d$ and $q \not \mid r$ we have

$$\begin{aligned} B_{k+1}\left( \frac{a}{r}+d\right) - B_{k+1}\left( \frac{a}{r}\right) \equiv d \cdot B_{k+1}^\prime (a/r) \pmod {q^2}. \end{aligned}$$

The lemma follows from (6) and Lemma 2.1. $\square$

#### Lemma 2.3

Let *k*, *r* be integers with $k \ge 2$ and $r \ne 0$. Let $q \ge k+3$ be a prime not dividing *r* such that the congruence $B_k(x) \equiv 0 \pmod {q}$ has no solutions. Let *d* be a positive integer such that ${{\mathrm{\upsilon }}}_q(d)=1$. Then Eq. (2) has no solutions (i.e. $d \notin \mathcal {A}_{k,r}$).

#### Proof

Suppose $(x,y,n)=(a,b,n)$ is a solution to (2). By Lemma 2.2,

$$\begin{aligned} r^k \cdot d \cdot B_k(a/r) \equiv b^n \pmod {q^2}. \end{aligned}$$

However, the hypotheses of the lemma ensure that the left-hand side has *q*-adic valution 1. Thus ${{\mathrm{\upsilon }}}_q(b^n)=1$ giving a contradiction. $\square$

#### Remarks

- For $k \ge 3$ odd, the *k*-th Bernoulli polynomial has known rational roots 0, 1 / 2, 1. Thus the criterion in the lemma fails to hold for all primes *q*. For even $k \ge 2$ we shall show that there is a positive density of primes *q* such that $B_k(x)$ has no roots modulo *q*.
- The second Bernoulli polynomial is $B_2(x)=x^2-x+1/6$. By quadratic reciprocity, this has a root modulo $q \not \mid 6$ if and only if $q \equiv \pm 1 \pmod {12}$. We thus recover the result of Bai and Zhang mentioned in the introduction: if $q \equiv \pm 5 \pmod {12}$ and ${{\mathrm{\upsilon }}}_q(d)=1$ then (1) has no solutions with $k=2$.

## A Galois property of even Bernoulli polynomials

### Proposition 3.1

Let $k \ge 2$ be even, and let $G_k$ be the Galois group of the Bernoulli polynomial $B_k(x)$. Then there is an element $\mu \in G_k$ that acts freely on the roots of $B_k(x)$.

There is a long-standing conjecture that the even Bernoulli polynomials are irreducible; see for example [3, 4, 14]. One can easily deduce Proposition 3.1 from this conjecture. We give an unconditional proof of Proposition 3.1 in Sect. 5. As noted previously, if *k* is odd, then $B_k(x)$ has rational roots 0, 1 / 2, 1, so the conclusion of the proposition certainly fails for odd *k*.

### A density result

Let $\mathcal {A}$ be a set of positive integers. For *X* positive, define

$$\begin{aligned} \mathcal {A}(X)=\# \{d \in \mathcal {A}: d \le X \}. \end{aligned}$$

The *natural density* of $\mathcal {A}$ is defined as the limit (if it exists)

$$\begin{aligned} \delta (\mathcal {A})=\lim _{X \rightarrow \infty } \frac{\mathcal {A}(X)}{X}. \end{aligned}$$

For a given prime *q*, define

$$\begin{aligned} \mathcal {A}^{(q)}=\{d \in \mathcal {A}\; : \; {{\mathrm{\upsilon }}}_q(d)=1\}. \end{aligned}$$

We shall need the following result of Niven ([13] Corollary 1).

#### Theorem 3

(Niven) Let $\{q_i\}$ be a set of primes such that $\delta (\mathcal {A}^{(q_i)})=0$ and $\sum q_i^{-1}=\infty$. Then $\delta (\mathcal {A})=0$.

### Proposition 3.1 implies Theorem 1

We now suppose Proposition 3.1 and use it to deduce Theorem 1. Let $k \ge 2$ be an even integer. Write $G_k$ for the Galois group of the Bernoulli polynomial $B_k(x)$. Let $\mu \in G_k$ be the element acting freely on the roots of $B_k(x)$ whose existence is asserted by Proposition 3.1. By the Chebotarev density theorem ([6] Chapter VIII) there is a set of primes $\{q_i\}_{i=1}^\infty$ having positive Dirichlet density such that for each $q=q_i$, the Frobenius element ${{\mathrm{Frob}}}_q \in G_k$ is conjugate to $\mu$. We omit from $\{q_i\}$ (without affecting the density) the following:

- primes $$q \le k+2$$;
- primes *q* dividing *r*;
- primes *q* dividing the numerator of the discriminant of $B_k(x)$ (which is non-zero by Theorem 2).

As $\mu$ acts freely on the roots of $B_k(x)$, it follows that the polynomial $B_k(x)$ has no roots modulo any of the $q_i$. Now let $\mathcal {A}=\mathcal {A}_{k,r}$ be as in the statement of Theorem 1. By Lemma 2.3, if ${{\mathrm{\upsilon }}}_{q_i}(d)=1$ then $d \notin \mathcal {A}$. It follows that $\mathcal {A}^{(q_i)}=\emptyset$. By Theorem 3, we have $\delta (\mathcal {A})=0$ as required.

## The 2-adic Newton polygons of even Bernoulli polynomials

### Lemma 4.1

Let $k \ge 2$ be even and write $k=2^s t$ where *t* is odd and $s \ge 1$. The 2-adic Newton polygon of $B_k(x)$ consists two segments:

(i) a horizantal segment joining the points $(0,-1)$ and $(k-2^s,-1)$;
(ii) a segment joining the points $(k-2^s,-1)$ and (*k*, 0) of slope $1/2^s$.

### Proof

Consider the definition of $B_k(x)$ in (3). We know that $B_0=1$, $B_1=-1/2$ and $B_m=0$ for all odd $m \ge 3$. From the von Staudt–Clausen theorem, we know that ${{\mathrm{\upsilon }}}_2(B_m)=-1$ for even $m \ge 2$. It follows that the Newton polygon is bounded below by the Horizontal line $y=-1$.

We shall need to make use of the following result of Kummer (see [11]): if *p* is a prime, and *u*, *v* are positive integers then

$$\begin{aligned} \left( {\begin{array}{c}u\\ v\end{array}}\right) \equiv \left( {\begin{array}{c}u_0\\ v_0\end{array}}\right) \left( {\begin{array}{c}u_1\\ v_1\end{array}}\right) \pmod {p}, \end{aligned}$$

where $u_0$, $u_1$ are respectively the remainder and quotient on dividing *u* by *p*, and likewise $v_0$, $v_1$ are respectively the remainder and quotient on dividing *v* by *p*. Here we adopt the convention $$\left( {\begin{array}{c}r\\ s\end{array}}\right) =0$$ if $r<s$. Applying this with $p=2$ we see that

$$\begin{aligned} \left( {\begin{array}{c}k\\ 2^s\end{array}}\right) = \left( {\begin{array}{c}2^s t\\ 2^s\end{array}}\right) \equiv \left( {\begin{array}{c}t\\ 1\end{array}}\right) \equiv t \equiv 1 \pmod {2}. \end{aligned}$$

Thus the coefficient of $x^{k-2^s}$ in $B_k(x)$ has 2-adic valuation $-1$. Since the constant coefficient of $B_k(x)$ also has valuation $-1$, we obtain the segment (i) as part of the Newton polygon. We also see that for $0<v<2^s$,

$$\begin{aligned} \left( {\begin{array}{c}k\\ v\end{array}}\right) \equiv 0 \pmod {2}, \end{aligned}$$

and so the valuation of the coefficient of $x^{k-v}$ is $\ge 0$. Finally the coefficient of $x^k$ is $B_0=1$ and so has valuation 0. This gives segment (ii) and completes the proof. $\square$

### Remarks

Inkeri [12] showed that $B_k(x)$ has no rational roots for *k* even. His proof required very precise (and difficult) estimates for the real roots of $B_k(x)$. Lemma 4.1 allows us to give a much simpler proof of the following stronger result.

### Theorem 4

Let *k* be even. Then $B_k(x)$ has no roots in ${\mathbb Q}_2$.

### Proof

Indeed, suppose $\alpha \in {\mathbb Q}_2$ is a root of $B_k(x)$. From the slopes of the Newton polygon segments we see that ${{\mathrm{\upsilon }}}_2(\alpha )=0$ or $-1/2^s$. As ${{\mathrm{\upsilon }}}_2$ takes only integer values on ${\mathbb Q}_2$, we see that ${{\mathrm{\upsilon }}}_2(\alpha )=0$ and so $\alpha \in {\mathbb Z}_2$. Let $f(x)=2B_k(x) \in {\mathbb Z}_2[x]$. Thus $f(\alpha )=0$ and so $f(\overline{\alpha })=\overline{0} \in {\mathbb F}_2$. However, $\overline{\alpha } \in {\mathbb F}_2=\{\overline{0},\overline{1}\}$. Now $f(\overline{0})=\overline{(2B_k)}=\overline{1}$, and from (4) we know that $f(\overline{1})=f(\overline{0})=\overline{1}$. This gives a contradiciton. $\square$

Although Theorem 4 is not needed by us, its proof helps motivate part of the proof of Proposition 3.1.

## Completing the proof of Theorem 1

### A little group theory

#### Lemma 5.1

Let *H* be a finite group acting transitively on a finite set $\{ \beta _1,\cdots ,\beta _n\}$. Let $H_i \subseteq H$ be the stabilizer of $\beta _i$, and suppose $H_1=H_2$. Let $\pi \; : \; H \rightarrow C$ be a surjective homomorphism from *H* onto a cyclic group *C*. Then there is some $\mu \in H$ acting freely on $\{\beta _1,\cdots ,\beta _n\}$ such that $\pi (\mu )$ is a generator of *C*.

#### Proof

Let $m=\#C$ and write $C=\langle \sigma \rangle$. Consider the subset

$$\begin{aligned} C^\prime =\{ \sigma ^r \; : \; \gcd (r,m)=1\}; \end{aligned}$$

this is the set of elements that are cyclic generators of *C*, and has cardinality $\varphi (m)$, where $\varphi$ is the Euler totient function. As $\pi$ is surjective we see that

7 $$\begin{aligned} \# \pi ^{-1}(C^\prime )=\frac{\varphi (m)}{m} \cdot \# H. \end{aligned}$$

As *H* acts transitively on the $\beta _i$, the stabilizers $H_i$ are conjugate and so have the same image $\pi (H_i)$ in *C*. If this image is a proper subgroup of *C*, then take $\mu$ to be any preimage of $\sigma$. Thus $\pi (\mu )=\sigma$ is a generator of *C*, and moreover, $\mu$ does not belong to any of the stabilizers $H_i$ and so acts freely on $\{\beta _1,\cdots , \beta _n\}$, completing the proof in this case. Thus we suppose that $\pi (H_i)=C$ for all *i*. It follows that

8 $$\begin{aligned} \# \pi ^{-1} (C^\prime ) \cap H_i= \frac{\varphi (m)}{m} \cdot \# H_i = \frac{\varphi (m)}{m} \cdot \frac{\# H}{n}, \end{aligned}$$

where the second equality follows from the Orbit-Stabilizer Theorem. The lemma states that there is some element $\mu$ belonging to $\pi ^{-1}(C^\prime )$ but not to $\cup H_i$. Suppose otherwise. Then

$$\begin{aligned} \pi ^{-1}(C^\prime ) \subseteq \bigcup _{i=1}^n H_i, \end{aligned}$$

and therefore

9 $$\begin{aligned} \pi ^{-1} (C^\prime )=\bigcup _{i=1}^n \pi ^{-1}(C^\prime ) \cap H_i. \end{aligned}$$

Now (7), (8) and (9) together imply that the $\pi ^{-1}(C^\prime ) \cap H_i$ are pairwise disjoint. This contradicts the hypothesis that $H_1=H_2$ completing the proof. $\square$

*Proof of Propostion* 3.1 We now complete the proof of Theorem 1 by proving Proposition 3.1. Fix an even $k \ge 2$, and let *L* be the splitting field of $B_k(x)$. Let $G_k={{\mathrm{Gal}}}(L/{\mathbb Q})={{\mathrm{Gal}}}(B_k(x))$ be the Galois group of $B_k(x)$. Let $\mathfrak {P}$ be a prime of *L* above 2. The 2-adic valuation ${{\mathrm{\upsilon }}}_2$ on ${\mathbb Q}_2$ has a unique extension to $L_\mathfrak {P}$ which we continue to denote by ${{\mathrm{\upsilon }}}_2$. We let $H={{\mathrm{Gal}}}(L_\mathfrak {P}/{\mathbb Q}_2) \subseteq G_k$ be the decomposition subgroup corresponding to $\mathfrak {P}$.

From Lemma 4.1 we see that $B_k(x)$ factors as $B_k(x)=g(x)h(x)$ over ${\mathbb Q}_2$ where the factors *g*, *h* correspond respectively to the segments (i), (ii) in the lemma. Thus *g*, *h* have degree $k-2^s$ and $2^s$ respectively. We denote the roots of *g* by $\{\alpha _1,\ldots ,\alpha _{k-2^s}\} \subset L_\mathfrak {P}$ and the roots of *h* by $\{\beta _1,\ldots ,\beta _{2^s}\} \subset L_\mathfrak {P}$. From the slopes of the segments we see that ${{\mathrm{\upsilon }}}_2(\alpha _i)=0$ and ${{\mathrm{\upsilon }}}_2(\beta _j)=-1/2^s$. It clearly follows that *h* is irreducible and therefore that *H* acts transitively on the $\beta _j$. Moreover, from the symmetry (4) we see that $1-\beta _1$ is a root of $B_k(x)$, and by appropriate relabelling we can suppose that $\beta _2=1-\beta _1$. In the notation of Lemma 5.1, we have $H_1=H_2$. Now let $C={{\mathrm{Gal}}}({\mathbb F}_\mathfrak {P}/{\mathbb F}_2)$, where ${\mathbb F}_\mathfrak {P}$ is the residue field of $\mathfrak {P}$. This group is cyclic generated by the Frobenius map: $\overline{\gamma } \mapsto \overline{\gamma }^2$. We let $\pi \; : \; H \rightarrow C$ be the induced surjection. By Lemma 5.1 there is some $\mu \in H$ that acts freely on the $\beta _i$ and such that $\pi (\mu )$ generates *C*. To complete the proof of Proposition 3.1 it is enough to show that $\mu$ also acts freely on the $\alpha _i$. Suppose otherwise, and let $\alpha$ be one of the $\alpha _i$ that is fixed by $\mu$. As ${{\mathrm{\upsilon }}}_2(\alpha )=0$, we can write $\overline{\alpha } \in {\mathbb F}_\mathfrak {P}$ for the reduction of $\alpha$ modulo $\mathfrak {P}$. Now $\alpha$ is fixed by $\mu$, and so $\overline{\alpha } \in {\mathbb F}_\mathfrak {P}$ is fixed by $\langle \pi (\mu ) \rangle =C$. Thus $\overline{\alpha } \in {\mathbb F}_2$ and so $\overline{\alpha }=\overline{0}$ or $\overline{1}$. Now let $f(x)=2 B_k(x) \in {\mathbb Z}_2[x]$. Thus $f(\overline{\alpha })=\overline{0}$. But $f(\overline{0})=\overline{(2B_k)}=\overline{1}$, and from (4) we know that $f(\overline{1})=f(\overline{0})=\overline{1}$. This contradiction completes the proof.
